# Numerical Simulation on the Dissociation, Formation, and Recovery of Gas Hydrates on Microscale Approach

**DOI:** 10.3390/molecules26165021

**Published:** 2021-08-19

**Authors:** Mar’atus Sholihah, Wu-Yang Sean

**Affiliations:** Department of Environmental Engineering, Chung Yuan Christian University, Chung-Li, Taoyuan 32023, Taiwan; mara1004tus@gmail.com

**Keywords:** gas hydrate, microscale porous media, dissociation, formation, recovery

## Abstract

Investigations into the structures of gas hydrates, the mechanisms of formation, and dissociation with modern instruments on the experimental aspects, including Raman, X-ray, XRD, X-CT, MRI, and pore networks, and numerical analyses, including CFD, LBM, and MD, were carried out. The gas hydrate characteristics for dissociation and formation are multi-phase and multi-component complexes. Therefore, it was important to carry out a comprehensive investigation to improve the concept of mechanisms involved in microscale porous media, emphasizing micro-modeling experiments, 3D imaging, and pore network modeling. This article reviewed the studies, carried out to date, regarding conditions surrounding hydrate dissociation, hydrate formation, and hydrate recovery, especially at the pore-scale phase in numerical simulations. The purpose of visualizing pores in microscale sediments is to obtain a robust analysis to apply the gas hydrate exploitation technique. The observed parameters, including temperature, pressure, concentration, porosity, saturation rate, and permeability, etc., present an interrelationship, to achieve an accurate production process method and recovery of gas hydrates.

## 1. Introduction

Gas hydrates are components, such as ice crystals, configured from water molecules under high-pressure and low-temperature conditions, from the bonding interaction between the confined water molecules with guest molecules and hydrogen bonds in the water molecule structures [1,2]. Natural gas hydrates (NGH) are a promising energy source. Several studies have looked into gas production from natural gas hydrate reservoirs as renewable energy resources [3,4,5].

Methane hydrates (MH) are natural gas hydrates, abundantly obtainable in sediment hydrates, and are currently being explored [6,7,8,9]. With an enormous global resource volume of about 3000 trillion m^3^, and a high-energy storage capacity of 170 CH_4_ *v*/*v* of methane hydrate, energy recovery from natural methane hydrate is technically feasible and economically viable [10]. Research on NGH exploitations involve production and recovery of gas hydrates carried out by several approaches, i.e., numerical simulations, and experimental and field trial exploitation for different technologies [3,11].

One of the most challenging problems is producing stable methane hydrates from seabed located in complex flows and transportations, mostly in unconsolidated porous layers [12,13]. Therefore, insight into MH formation and dissociation processes is essential to establish appropriate technologies for safe and efficient energy recovery and production [14].

Considerable works have been conducted throughout the years, involving experimental and numerical research on the dissociation [3,6,15,16] and formation of methane hydrates [14,16]. Numerous methods, such as thermal stimulation, depressurization, inhibitor injection, or a combination of these methods, have been promoted; they refer to the breakdown of the thermodynamic equilibrium of the gas molecule [6,14,17,18]. In order to assess a prospective method of feasible gas production or exploitation from hydrate reservoirs, various data are needed to examine the dissociation and formation process in porous media, including saturation, porosity, and permeability [3,19,20,21]. Besides gas production of methane from the extensive hydrate resource, an attempt to sequester CO_2_ in porous sediments is also concerning, due to the massive potential of a safe CO_2_ mitigation method [9,22,23].

An experimental approach can present essential parameters for the exploration and exploitation of gas hydrates. In recent years, the modern analytical instruments and techniques on the experimental aspects, including Laser Raman spectroscopy (Raman), X-ray diffraction (XRD), X-ray computed tomography (X-CT), scanning electron microscope (SEM), nuclear magnetic resonance (NMR), high pressure differential scanning calorimetry (DSC), were conducted in the study of gas hydrate structure, formation mechanisms, and phase equilibrium [24,25,26,27,28,29]. However, using the experimental aspect, there was difficulty exploring some critical information (involving gas behavior), such as evaluating the state of porous medium channels during methane hydrate dissociation, due to inadequate experimental equipment, such as a lack of accurate measurements or observations on the experimental equipment capacity, unrepresentative experimental conditions, for example, due to samples not fitting to the real field, or damaged original field samples, human error, and other variables [15,25,26,30]. Hence, numerous studies have conducted modeling or numerical analysis to complete the relevant factors for the exploitation of gas hydrates.

One numerical simulation that has potential (when investigating the conditions of the gas hydrate complexes) is the Lattice Boltzmann Method (LBM). LBM can solve the coupled multiple process problems due to its capacity in computing the complex geometrical boundaries [31]. Considering gas hydrate conditions for dissociation and formation are multi-phase and multi-component complexes, particular attention should be paid to the microscale effects of the gas hydrate processes in the microporous media channels [7].

In addition, the molecular dynamics (MD) simulation was implemented as a robust method to overcome microporous media modeling by studying the performances of the different systems and chemicals at the molecular stages [15,32]. Nowadays, MD simulation is carried out to investigate the radial distribution function (RDF), mean square displacement (MSD), thermal expansion coefficient, diffusion coefficient, heat capacity, and thermal conductivity for different structures of gas hydrates at the various processes and thermodynamic conditions [33,34,35].

Therefore, comprehensive research is needed to better understand the processes and mechanisms involved, including hydrate dissociation conditions, hydrate formation, hydrate recovery in microscale porous media, emphasizing micro-modeling experiments, and 3D imaging [4]. The purpose of visualization of pores in sediments on a microscale is to obtain a robust analysis of the characterization of the physical structure of marine sediments, to model complex processes in sediment pores, such as the phenomenon of multi-phase fluid flow and particle transport in applying gas exploitation techniques.

## 2. Natural Gas Hydrate in the Sediment

Natural gas hydrates (NGHs) are ice-like crystalline solids, consisting of water and natural gas, referred to as burning ice. High pressure and low temperature conditions are needed to form gas hydrates. The process occurs when water molecules are connected to each other in an open structure and encage gas molecules to stabilize the clathrate structure [2]. The trapped gas molecules are dominated most by methane—abundant in nature with attractive energy potential [36]. Moreover, the affluent availability of gas hydrates around the globe make them promising options as fuel resources [37].

**Figure 1:**
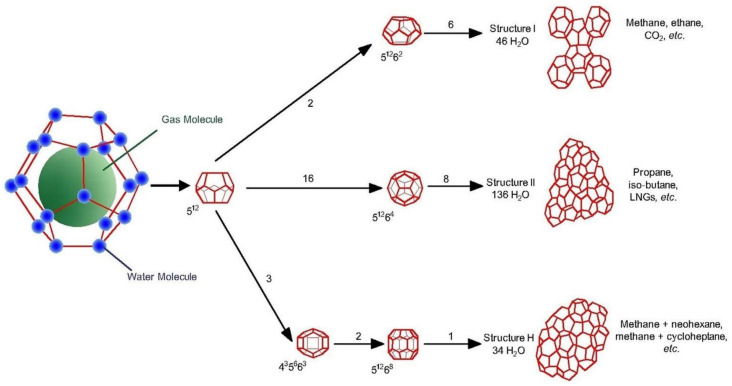
Structure of natural gas hydrate [38].

Hydrate structures consist of three known structures; structure 1 (sI), structure 2 (sII), and structure H (sH), as shown in Figure 1 [38]. Each structure has a distinct set of water molecule cages that form a unit cell. The existence of any structure depends on crystal lattice parameters, van der Waals force, and free diameters of cavities. Thus, gas hydrate crystal structures are linked to their formation.

The cubic sI clathrate can only accommodate small-sized hydrocarbon molecules, such as methane and ethane. It is the vastest spread structure in nature. Besides, sI can hold non-hydrocarbon molecules, such as CO_2_ and H_2_S [38]. The sII clathrate can accommodate both small hydrocarbon molecules, such as C1, C2, propane (C3), and isobutene (i-C4). On the other hand, sH clathrate called “large cage” can even hold molecules with significantly more diameter than isobutene (i-C4), such as i-C5 [39]. In general, sII and sH are more stable than sI [38].

Approximately 99% of the NGHs are found in oceanic sediments and remains in onshore artic environments [40]. The NGH exploitation is generally emphasized on the disturbance of the thermodynamic equilibrium of the hydrates, via depressurization, thermal stimulation, and inhibitor injection, implemented independently or combined [14].

## 3. Modeling Regarding Dissociation and Formation of Gas Hydrate

Gas hydrate production on a commercial scale, from natural hydrates, remains challenging, due to poor understanding of hydrate–host sediment interactions for its existence under low-temperature and high-pressure conditions [41]. Simulation modeling has been widely used based on technology breakthroughs to design, operate, control, and optimize the processes of exploitation and recovery of natural gas hydrates [42].

One of the main barriers in exploiting gas hydrates is hydrate reformation, which needs to be handled adequately [14]. Hydrate dissociation involves an endothermic process, which is strongly dependent on external energy supply. When the energy supply is inadequate, the temperature will decrease during the hydrate dissociation process, to lead the hydrate reformation [43]. Various methods have been proposed to assure safety and efficiency in gas hydrate exploitation, including CH_4_ gas leakage and wellbore blockages due to hydrate reformation or ice formation [7]. According to Wang, Dong [30], the hydrate dissociation stage has the potential to form ice, which could inhibit the dissociation process due to the thickened ice. Variations of the pressure may indicate the possibility of hydrate reformation or deformation in porous media. Moreover, heat change is a crucial factor for hydrate dissociation or reformation; it shows a complex change as a result of the ice formation or melting.

Furthermore, many studies have focused on the formation of carbon dioxide. Carbon capture and storage (CSS) is a credible technology used to mitigate CO_2_ emissions [44,45,46,47]. There is the potential of a blockage pipe during CCS. Nakashima and Sato [48] performed the formation of CO_2_ hydrates by injecting CO_2_ into the underwater sand sediments under high pressure and low temperature conditions. However, the formation of these hydrates can reduce the permeability, which has the potential to block the gas flow. It is important to ensure that the injected gas expands over a large area, while maintaining sufficient permeability to maximize the sequestration space. Another study based on Yu, Sato [49] investigated the blockages by predicting the morphology of sediment hydrate formation under gas–liquid two-phase flow conditions. The simulation results showed that the formation of CO_2_ hydrates (in the hydrate film behind the gas front) contributes greatly to the saturation of CO_2_ hydrates near the boundary, thereby occupying the pore space of the sand sediments, resulting in reduced permeability and blocking the gas flow. Takahashi, Sato [50] noted that the formation of gas hydrates on the interface between gas and water consists of two stages: gas diffusion through the CO_2_-hydrate film and the consequent CO_2_-hydrate formation on the interface between the film and water. It also leads to a new reaction interface, which is part of the interface between the gas and aqueous phases and is not covered with CO_2_-hydrate. Zatsepina and Pooladi-Darvish [51] note that adjusting the injected CO_2_ temperature could evade the hydrate formation in the production pipeline, which often has formation clogging issues. Their results show that, at the top and bottom, more hydrates are formed. The hydrate formation heat vanishes to the cap and bedrock. Eventually, the CO_2_ hydrate quantity is many times greater than the original gas, in-situ. Apart from managing the injection rate, the presence of gaseous hydrocarbon, in-situ, or injected impurities, CO_2_ and reservoir cooling after the CO_2_ injection process, could intensify CO_2_ storage capabilities in depleted gas pools. Besides the blockage pipe, CO_2_ leakage in hydrates is also considered. However, the probability of that incident is immensely low, such as a massive earthquake or other catastrophes [47]. If there is a CO_2_ leak on the seabed, it will lower the pH of the seawater and can affect the marine ecosystem [44]. Therefore, to predict the sealing potential of the CO_2_ hydrate, it is necessary to observe the effective permeability of the sediment after the CO_2_ hydrate is formed [23].

In addition to blockages and leakages, an important factor to predict the gas production rate is the surface area of the gas hydrates [16,52,53]. Nakayama, Ogasawara [52] proposed a model equation for the hydrate-specific surface area, by comparing the temporal change of the measured total gas production and its rate. Similarly, Ruan and Li [53] also observed the surface area of methane hydrate by comparing experimental and numerical simulation results during hydrate dissociation. The conceptual model for the hydrate dissociation surface area is proposed based on the hydrate morphology, which is related to the surface area of hydrate dissociation with the porosity, saturation, and average diameter of the sand sediment particles. They discovered that the proposed model formula (for the hydrate dissociation surface area, presenting the grain coating surface area) modeled very well at low hydrate saturation, while the high hydrate saturation was suitable for the pore-filling surface area. In addition, it was stated that the surface area of hydrate dissociation had a significant impact on cumulative gas production.

Development of microscale devices integrated with modern analytical technologies have been widely carried out, including laser Raman spectroscopy (Raman), X-ray diffraction (XRD), X-ray computed tomography (X-CT), scanning electron microscope (SEM), nuclear magnetic resonance (NMR), high pressure differential scanning calorimetry (DSC); these were conducted in the study of gas hydrate structures, formation mechanisms, and phase equilibrium [24,25,26,27,28,29].

Using the MRI and X-ray computed tomography (X-ray CT), the main factors controlled are the nucleation and growth of hydrates in the pore spaces of the sediments, microscopically [24]. Whereas Raman spectroscopy, X-ray diffraction (XRD), and nuclear magnetic resonance (NMR) are recognized as the three main methods used to evaluate the microstructures of gas hydrates [24]. Raman spectroscopy is the most prevalent way to identify hydrate structures, formation and dissociation kinetics, and even gas storage. Meanwhile, XRD is effectively used to determine the hydrate crystal structure. Moreover, NMR is commonly applied to the study of multi-component gas hydrates. It has a high sensitivity in observing guest molecular dynamics of various types of gas hydrates, so it can be used to study the complex and multicomponent structures of gas hydrates [24,25,26].

Raman is a spectroscopic technique used to investigate the chemical bonds of guest molecules and water molecules to obtain the configuration of the hydrate [24]. Zhang, Du [25] reported the discovery of gas hydrates on the South China Sea by investigating the in-situ chemical configurations and cage structures of hydrates using Raman. It is stated that natural hydrate deposits, especially those in early formation stages, are not monolithic single structures. However, they exhibit significant small-scale heterogeneities due to the inclusion of free gas and surrounding seawater. The inclusions also serve as indicators of possible mechanisms of hydration formation. Truong-Lam, Cho [54] observed the formation and dissociation processes using in-situ Raman. They mentioned that, at the beginning of the hydrate growth phase, the Raman peak intensity of methane (occupying large and small cages) increased after a sudden drop of dissolved methane. Thereto, the large cage encapsulation rate was faster than the small cage rate during the formation of hydrates.

Moreover, Liu, Meng [55] demonstrated an exploitation gas hydrate in the Pearl River Mouth (PRM) basin using Raman and XRD. Their results showed that the typical structure 1 (sI) with methane cage occupancy was more than 99.5% in large cages and 91.4% in small cages, according to the hydration number of 5.90 by thermodynamic calculations. The molecular composition of the hydrate bound gas indicated that the guest molecule was predominantly methane (>99.9%), with trace amounts of ethane (0.04%) and propane (0.01%). Liu, Meng [26] performed a systematic analysis of modern instruments, including laser grain size analyzers, X-ray CT, scanning electron microscopy (SEM), XRD, Raman spectroscopy, gas chromatography, and isotope mass spectrometry, on hydrate-carrying sediment samples found from the Shenhu area of the South China Sea in 2016. Their result showed that the sediment is much more refined than that recovered from the Shenhu area in 2007. Based on Raman data and XRD analysis, gas hydrates exhibited a typical structure 1 (sI) hydrate, with methane molecule occupancy higher than 99.3% in large cages and 81.5–91.4% in small cages, respectively. The crystal lattice parameter was 11.89 A with a hydration number of about 6.0. Methane was clearly the dominant component, ranging from 97.6% to 99.95%.

X-ray computed tomography (CT) and magnetic resonance imaging (MRI) was recently used to visualize MH dissociation in porous media. Fan, Sun [56] performed MRI visualization and analysis for the dissociation of methane hydrate by controlling the depressurization rate gradually to production pressure to avoid ice formation or hydrate reformation during the depressurization process. MH dissociates spatially at the onset of depressurization. Low production pressure can supply more sensible heat, and MH dissociates rapidly under low production pressure. Due to insufficient heat transfer from the surrounding thermostatic bath, a large amount of ice is generated spatially. Then ambient heat transfer promotes ice melting and dissociation of hydrates from the surrounding walls. In addition, Lv, Jiang [57] presented the formation and dissociation of CH_4_ hydrate using MRI to understand the pore habits of CH_4_ hydrate and gas seepage behavior in hydrate-bearing sediment (HBS). The results showed that homogeneous sand packs of CH_4_ hydrates are preferably formed in the lowest water saturation. For heterogeneous sand packs, the hydrate nucleation occurs in small grain sizes, and the final hydrate saturation is the highest. In addition, the dissociation process of CH_4_ hydrate consisted of three stages—the initiation stage, the rapid dissociation stage, and the post dissociation stage. In addition, Almenningen, Gauteplass [58] visualized CO_2_-water drainage flow followed by hydrate formation using high-field MRI. It is important to identify CO_2_ flow and hydrate growth patterns in sediment pores in order to obtain an effective hydrate sealing process. The image results interpret the nucleation of the hydrate at the liquid–liquid interface, whereas the aqueous phase occurs only due to the presence of dissolved CO_2_. Furthermore, the growth pattern of the pore-filling hydrates effectually lowers the permeability of the sandstone and demonstrates the potential of CO_2_ hydrates as a sealing process during CO_2_ sequestration.

As discussed above, effective permeability is a critical factor to predict gas productivity from CH_4_ hydrate in underwater sand sediments as it affects the hydrate distribution [19,59]. Permeability is an important characteristic in hydrate-bearing porous media to investigate the mobility of CH_4_ and water can pass through the pore structure [3,45,60]. The permeability variation caused by hydrate dissociation will allow for further understanding on hydrate exploitation or gas production [19,20,59,61,62]. Chen, Yamada [59] used thermal stimulation and found that the dissociation time is 2.23 times and 7.02 times longer when the permeability is lowered. They also suggested that suitable warm-up and increased permeability should be applied for an effective dissociation process based on the temperature and permeability results. According to Gao and Li [63], by describing the effect of effective stress and water saturation on the relative permeability of gases in tight sandstones, the presence of water, and the effective stress, increased. The permeability jail concept could be used to present field observations under the conditions of little water or gas production. Since the relative permeability of the gas phase gives the opposite result to water, hydraulic fracturing without water has great potential in effectively unlocking tight gas resources.

As mentioned above, the permeability of HBS is one of the essential factors in observing the behavior and distribution of the fluid, as well as gas performance during hydrate exploitation. The wettability of porous media dramatically influences the relative permeability of two phases (gas–water) [64,65,66]. In addition, the wettability of HBS is a critical factor for accurate numerical simulations to investigate multi-phase flow prediction and seepage behavior in porous media in hydrate sediment, including CO_2_ sequestration, gas transportation, and storage [67]. In accordance with Wang, Zhao [64] investigated the effects of the wettability of hydrate-bearing porous media on their seepage properties using a pore network model combined with X-ray computed tomography (X-ray CT). The simulation results showed that increasing the contact angle reduced the wettability of hydrate-bearing porous media, thereby increasing the relative permeability of the water phase and decreasing the gas phase of relative permeability at certain water saturation. Furthermore, the study authors noted that the relative permeability of the gas–water phase was influenced by the increases in the diameter of quartz sand particles. In addition, increased wettability and connectivity could reduce the residual gas saturation. Finally, it highly affects capillary pressure.

X-ray CT is one of the most reliable techniques to apply in examining the process of dynamics in-situ and exploring the formation and dissociation mechanisms of gas hydrates in the sediment matrices [21,45,68,69,70,71]. Moreover, micro-CT imaging analysis presents essential information for establishing an accurate numerical model to observe the permeability and other seepage factors of hydrate-bearing porous media [64,72,73]. Han, Kwon [70] utilized an X-ray CT to investigate the mechanism of fine migration of HBS during depressurization. They found that fine migration was more prominent in the coarse sands and with silty fine grains. Finally, depressurization of HBS can cause fine particle movement (migration), which affects the fluid flow behavior in the sediments. These factors will influence the long-term hydrocarbon production and wellbore stability, or may reduce sediment strength [17,70].

In some research cases, observations were conducted on hydrates stored in microstructure sediments under the seabed or rock reservoirs [72]. X-ray CT imaging methods can be used to accurately investigate the complex processes of formation, dissociation, and migration of hydrate [74]. Furthermore, the color difference phase separation approach can be used to separate the hydrate phase from the pore phase and grain phase [8,41,75]. Zhao and Zhou [8] conducted X-ray CT imaging experiments to evaluate the formation, migration, and dissociation of hydrates in sand pack specimens, at a microscale. The results indicate that accumulated hydrates migrated over time increases, and the gas hydrate ratio decreases by increasing the depth. In addition, the gas hydrates gradually accumulate in the pore phase with the growth of the hydrates, and the hydrates increase to a certain saturation. Then, due to the continuous growth of the hydrates, there will be a change from gas hydrates to the water hydrates. Therefore, it can be concluded that increases in relative porosity, relative pore size, and relative grain size can increases hydrate saturation due to the gas hydrate conversion to water hydrate. In addition, permeability may decrease while the hydrate phase in porous sediments increases due to occupation of the pore space by the hydrates. Moreover, Gao-Wei, Cheng-Feng [75] observed the in-situ pore scale of the gas hydrate distribution directly during the hydrate formation and dissociation process. In the first phase of hydrate formation, most of the processes are sediment particles connected to each other by hydrates or cementing models. In the next phase, the hydrates are typically shaped like floating or contact models. Since saturation increases, the floating hydrates begin to fuse with each other, and then the sediment particles cement again. On the other hand, it is indeed necessary to study the evolution of pore structures and fluid flow properties in HBS, which can be implemented by X-ray CT and pore network models [72,76,77,78,79].

**Figure 2:**
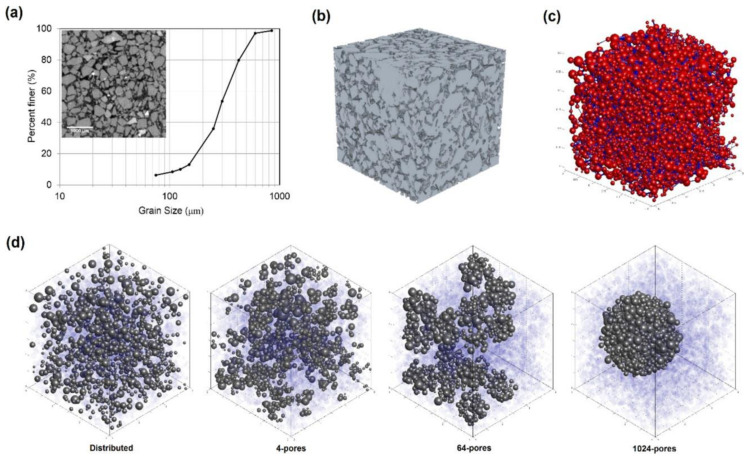
Pore-network modeling of hydrate distribution. (**a**) Specimen grain size distribution. (**b**) The 3D pore space was presented by µCT scan. (**c**) Pore network model. (**d**) Various hydrate morphologies for hydrate saturations (S_h_ = 0.2). It was clarified that the model consists of hydrate pores (gray color) and water pores (transparent blue color) [79].

**Figure 3:**
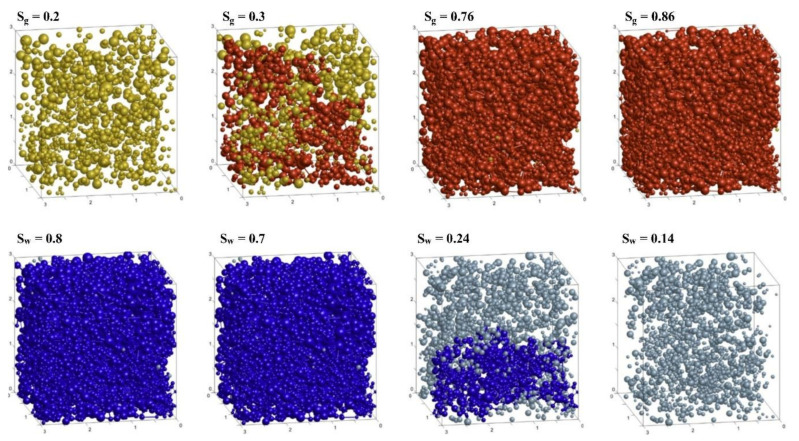
Illustrations of gas and water flow behavior during gas expansion in sediment hydrates under the dissociation process. The gas pores forming conductivity paths are represented in red, and the isolated gas pores are interpreted as yellow. Water pore forming conductivity paths are colored blue, and isolated water pores are colored light blue [79].

Mahabadi, Dai [79] applied the pore network extracted from micron-resolution CT images to various morphologies and hydrate saturations, as shown in Figure 2 and Figure 3. Then the processes of gas invasion, gas expansion, hydrate dissociation, and gas and water permeability were simulated. The results showed that excellent hydrate saturation pursued higher gas inlet pressure, higher residual water saturation, as well a steeper water retention curve. Increasing hydrate saturation allows gas permeability to decrease, but has marginal effects on water permeability in sediments with homogenously distributed hydrates. Under relative permeability conditions, it has impacts that are more apparent on hydrate morphology than hydrate saturation. Meanwhile, sediments with heterogeneously distributed hydrates tend to produce lower residual water saturation but higher gas and water permeability.

A pore network model combined with X-ray CT was also applied to evaluate the index properties and percolation characteristics of hydrate-bearing sediments [60,80,81]. According to Wang, Zhao [60], with the same level of water saturation, it was found that a larger porosity induced a larger relative permeability of the water phase but provided a smaller value of relative permeability of the gas phase. Wang, Zhao [80] declared that the hydrate saturation increases and the absolute permeability decreases. In addition, under the same water saturation, lower hydrate saturation leads to a larger relative permeability of the water phase, whereas it did not apply to gas relative permeability. Moreover, Wang, Wang [81] showed that the formation and dissociation of gas hydrates could lead to the change of pore structures and flow properties. An increase in gas hydrate saturation can induce a sharp decline in relative permeability of water, more immense irreducible water saturation, and a smaller gas–water percolation zone, while inversely proportional to the gas relative permeability. Finally, the gas hydrate dissociation will significantly affect the flow properties when compared to gas hydrate formation.

Since the application of the experimental approach is limited to exploring some important information on the complexity of real-scale condition surveys, it is necessary to develop numerical studies that can be joined and compared with experimental results to obtain more valid and accurate data [15,30]. By establishing numerical model for hydrate dissociation in porous media, gas production can be modeled and compared with the experimental data [6]. The hydrate dissociation process occurs by a heat and mass transfer process in porous media with a multi-phase flow and reaction. Computational fluid dynamics (CFD) is one numerical analysis technique that could be used to investigate the problem. Yu, Sean [82] established a pore-scale model by considering porosity by the CFD method and an unstructured grid to study the dissociation rate of methane hydrate inhomogeneous porous media. Application of the very thin layers (VTL) method is possible to calculate momentum, concentration, and thermal boundary layers by considering the porosity factor. It was found that, as the porosity increases, the flux increases due to the fast transport in large quantities. Indeed, the surface flux becomes saturated if the transport process in bulk flow is faster than the dissociation rate.

Similarly, Jeong, Chiang Hsieh [83] used the CFD method to establish a pore-scale model of surface dissociation of CO_2_ hydrates through Lagrangian periodic boundary conditions. In this study, the finite volume method (FVM) with unstructured mesh was constructed in a representative, regular, face-centered cubic unit. Observations were conducted by considering the surface mass transfer of CO_2_H and the heat transfer between the hydrate and water. The results indicated that the overall flux dissociation distribution was influenced by porosity in terms of water temperature. Thus, the higher water temperature can lead to higher dissociation flux at the hydrate surface.

Gas hydrate (i.e., methane) is a complex multi-component and multi-phase fluid flow process accompanied by mass transport and heat transfer in porous media [81]. One method that can be implemented to analyze that process is LBM [31,61]. Due to its attractive advantages, such as simplicity in coding, dealing with complex solid boundaries, and parallelization, LBM has become a very popular CFD method in many fields, such as multi-phase and multi-component flow, reactive flow, microscale, and nanoscale flow [66,84]. The micro-flow simulation was also conducted via the Lattice Boltzmann method (LBM) with pore-filling to evaluate the effect of hydrate on seepage characteristics in hydrate-bearing sediment grain-coating habits in porous media [61]. In work by Hou, Ji [61], to better understand the effects of hydrate on seepage properties in HBS, the micro-flow simulation was applied via LBM with pore-filling and grain-coating habits in porous media. The authors found that the mineral particle arrangement did not affect permeability variation. However, the permeability variation was sensitive to the habit of hydrate-formation habit and the morphology of hydrate-distribution morphology. According to Fuji, Kamada [44], microscopic computational domains consisting of sand grains and water-CO_2_ two phases, using LBM, were used to investigate the distribution of microscopic hydrates, which essentially controlled the effective permeability. The calculation results indicated the differences in the distribution of hydrates in the pore space, and the value of effective permeability depends on hydrate saturations, initial water saturation, and contact angles of water on the sand surface. In addition, it can be concluded that the difference in gas solubility has an impact on the difference in hydrate growth between CO_2_ and methane near the gas–water interface.

In addition, the reduction of permeability by quicksand blockage was, clearly, directly related to natural gas production [85]. Mitsuhori, Sato [86] simulated a numerical analysis using LBM coupled with micro-CT for the two-phase flow of solid water in a frame sand sediment. At first, the relatively larger particles were trapped by the frame sands, and then smaller particles filled the space between the larger particles. Hence, the mean size and the deviation as distribution factors are crucial parameters for the blockage. Zhang, Zhang [31] used LBM by combining the gas hydrate dissociation kinetic model, the single-phase flow LB model, the mass transport LB model, and the conjugate heat transfer LB model. The results showed that the simulation using LBM could easily evaluate the reactive transport framework effect to the coupled physicochemical thermal process and provide an understanding of the methane dissociation process at the pore scale. Endothermic reactions and heat transfer in porous media during the dissociation process result in temperature changes. Dissociation is accelerated with increasing inlet temperatures for both the pore-filling hydrate and the grain coating hydrate.

In addition to the studies discussed above, many studies have demonstrated the combination of LBM and X-rays in the two-phase flow [62,65,84,86,87,88,89]. According to Zhang, Kang [87], by simulating the gas distribution at different times, the high-density ratio accentuated the fingering phenomenon. As the density ratio increases, the displacement efficiency decreases. Moreover, as the density ratio increases, the gas saturation decreases in big pores and becomes zero in small pores or even in big pores followed by small channels. Meanwhile, due to the wettability of liquid, the residual liquid particularly distributes in the small pores and the edge of big pores. In addition, lowering the viscosity can be influential, increasing the recovery of the fluid.

**Figure 4:**
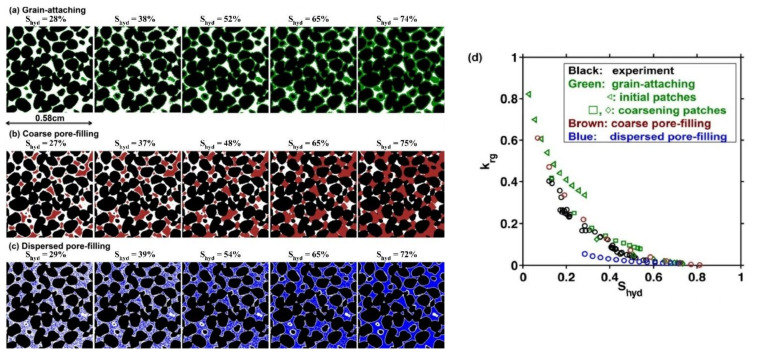
Hydrate-bearing sand model. (**a**) Grain-attaching. (**b**) Coarse pore-filling. (**c**) Dispersed pore-filling habits. (**d**) Gas relative permeability versus hydrate saturation data from the experimental and computational HBS [88].

Chen, Verma [88] estimated the relative permeability of gas (k_rg_) as a function of the hydrate saturation curve (S_hyd_) using digital models of hydrate-bearing sand based on the implementation of grain-attaching, coarse pore-filling, and dispersed pore-filling hydrate habits. The pore-scale measurements and modeling showed that the k_rg_-S_hyd_ curves were similar regardless of the hydrate crystal conditions. The k_rg_ data in hydrate-bearing sand could decrease (quite impressively) in the presence of porous hydrates, as shown in Figure 4.

Sand production greatly affects the reservoir stability and safety during gas hydrate exploitation in HBS. The formation and dissociation of trapped gas structures were affected by sand-bridge structures as a result of sand migration [85]. Yoshida, Yamaguchi [90] developed a numerical simulation method in the pore-scale computational domain to predict mud erosion caused by water flow. The simulation results showed that the erosion rate decreased to zero in less than 10 days under the conditions of an average velocity several times greater than the critical value for mud erosion. This can be inferred due to the surface of the mud with large shear stress eroded first, resulting in a large enough space in the pores of the sand grains, thereby slowing the water velocity to a value smaller than the critical value.

The molecular dynamics (MD) simulation is seen as an effective method to provide a molecular level of understanding on microscopic mechanisms, in regard to structural and dynamical properties [33,91,92,93]. The MD method is needed to understand the molecular structure of hydrates and the mechanism of dissociation or hydrate formation [15,34,94].

Yan, Li [92] observed the mechanism of methane hydrate dissociation by depressurization using MD. The concentration gradient between the H_2_O molecules in the hydrate surface layer and the inner layer forced the driving force of dissociation. The clathrates gradually collapse, and then the hydrates dissociate, layer-by-layer. On the other hand, it was also explained that the hydrate dissociation rate by depressurization was slower than the thermal stimulation and the inhibitor injection as hydrates have low thermal conductivity and temperature sensitivity [52,95]. Guo, Pan [95] used the MD simulation to simulate methane hydrate in a porous medium by considering the thermal conductivity. The authors noted that the thermal conductivity of methane hydrate increased with a temperature increase and grew faster near the freezing point. Moreover, thermal conductivity increases gradually under the same temperature as the decreases of the porous media pore sizes. It can be concluded that the different porous media pore sizes have a great effect on the thermal conductivity of the hydrates. Eventually, at the rapid increase in thermal conductivity, the dissociation process will begin immediately.

Moreover, MD was also used to investigate the mechanism/microscopic phenomena and intermolecular forces in the methane hydrate dissociation [96,97]. Kondori, Zendehboudi [33] observed the stability of the water cage at various dissociation times, temperatures, and pressures. They found that, based on the radial distribution function and the mean squared displacement of oxygen–oxygen and carbon–carbon atoms, the stability of the hydrate cage decreased with increasing temperature. However, with increasing cage occupancy and pressure, the hydrate stability also increased. Moreover, the addition of an inhibitor to a small cavity in the hydrate structure, such as methanol, can accelerate the dissociation of the hydrate by creating new hydrogen bonds between the water molecules and the inhibitor. In addition, Liu, Zhou [98] observed CO_2_ dissociation with inhibitors using MD. They found that the rate of dissociation of CO_2_ hydrate increased in the presence of two inhibitors, i.e., glycine, with a maximum concentration of 10% by weight and glucose, 1.2% by weight. Glycine aggregates on the solid–liquid surface of the initial structure, and the clathrate hydrate structure is wrecked due to -OH single bonds and -NH_2_ single bonds with water hydrogen bonds in the hydrate structure. Meanwhile, glucose has a ring-shaped structure and impaired functional groups, accelerating the hydrate dissociation due to the synergistic impact of steric hindrance. Yagasaki, Matsumoto [97] investigated the dissociation rate of methane with methanol and NaCl inhibitors. They found that the dissociation rate of methane hydrate increases with the formation of methane bubbles carried out by methanol and NaCl in the aqueous phase, because the bubbles absorb the surrounding methane molecules. However, the mechanisms of the two inhibitors are very different from each other. The bubbles from the NaCl solution increase the hydrophobic interactions between the methane molecules. In contrast, bubbles in methanol are formed due to it is amphiphilic.

Furthermore, a study on the constant energy of MD in the hydrate dissociation in contact with water was also carried out to investigate the role of mass and heat transfer in the dissociation rate [99,100,101]. According to Alavi and Ripmeester [100], under adiabatic conditions, the rate of dissociation of methane clathrate is influenced by heat and mass transfer from the breakdown of clathrate hydrate and methane gas discharge at the solid–liquid interface and the diffusion of methane through water. The results show that the temperature gradient between the clathrate and solution phases during the dissociation process is crucial, since it provides significant heat transfer. In addition, it turns out that the dissociation of clathrate does not occur gradually through the rupture of each cage but rather is integrated with a row of structure 1 cages parallel to the interface, and dissociates simultaneously [99,100]. Thus, huge amounts of methane gas are discharged close to the surface, and bubbles can form, which affects the mass transfer rate close to the clathrate phase surface.

In addition, MD has been considered a robust technique used to investigate crystal growth mechanisms [102,103], the solid/liquid and gas/liquid interfaces [104,105,106,107]. Several parameters were observed, such as potential energy changes, MSD of molecules, the number of methane molecules close to the solid/liquid interface, and the position of liquid/solid interfaces with time. Naeiji, Varaminian [105] demonstrated the kinetic growth of hydrate using MD. Their results showed that the potential energy and MSD of molecules in the layers close to the interfaces clearly reduced, indicating that the growth took place in these layers. Furthermore, the model can interpret the whole process of hydrate formation since the affinity as a driving force of the process exhibits that hydrate formation is a process that proceeds on a natural path. Naeiji, Varaminian [104] studied the different properties between methane/water and methane/water/hydrate systems. Their results indicated that the thermodynamic properties of the methane/water/hydrate system were lower than other systems, so the hydrate structure was more stable and reduced the system energy surface.

Moreover, the dissociation kinetics of CO_2_ hydrates were investigated by molecular dynamics (MD) [93,98,108]. Sarupria and Debenedetti [93] presented the results of a molecular dynamics study of the dissociation behavior of CO_2_ hydrates. The results showed that the dissociation rate depends on the fractional occupancy of each type of cage but is difficult to interpret in terms of overall hydrate occupancy. In particular, it was found that hydrates with overall occupancy depend on the emptying of large or small cages. For the same overall dwelling, small and large filled cages will dissociate more quickly with an empty large cage than with an empty small cage. Meanwhile, Zhang, Zhao [109] investigated the formation of CO_2_ hydrates by using MD; they stated that the most influential factors were temperature and molecular numbers. Hydrates are more likely to form in systems with a relatively large number of CO_2_ molecules and relatively low system temperatures.

## 4. Replacement Process of CH_4_ by Injection of CO_2_

The replacement of CH_4_ by CO_2_ in methane hydrates is a feasible way to achieve CH_4_ production and CO_2_ storage in the respective efforts of energy recovery and global warming mitigation [110,111,112,113,114,115]. Energy reserves from fossils continue to decrease, leading to the energy crisis and global warming [37,109,116,117,118,119]. Using CO_2_ to replace CH_4_ in hydrate sediments could either utilize CH_4_ or store CO_2_ as an embodiment of renewable energy and environmental protection [120,121]. As part of the replacement process, it is crucial to observe the formation of CO_2_ hydrates [51,122]. Research advances and numerical analyses in replacement processes that focus on laboratory studies have been carried out. The methods used to study the CH_4_–CO_2_ replacement processes are the same as in the formation and dissociation of hydrates, i.e., Raman spectroscopy, X-ray diffraction (XRD), magnetic resonance imaging (MRI), and X-ray CT [122,123,124]. While, numerical analysis can be simulated precisely using CFD and MD [89,94,125,126,127].

**Figure 5:**
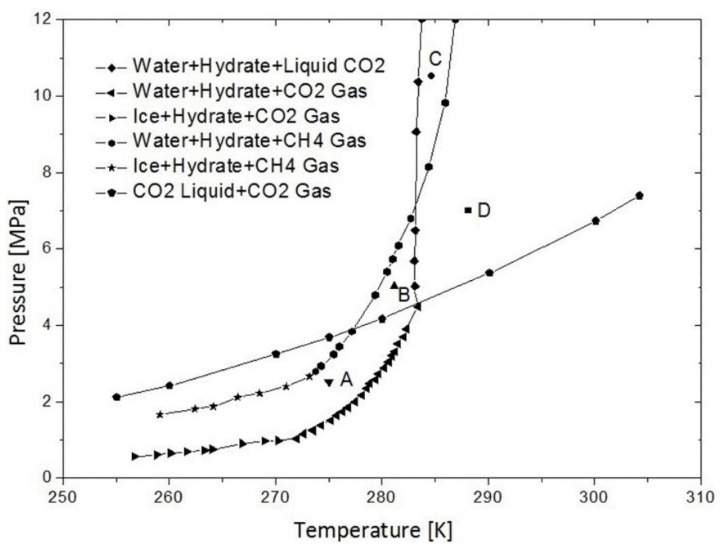
CH_4_ -CO_2_-H_2_O phase equilibrium diagram. Areas A, B, C and D indicate the possibility of phase changes for CO_2_ and CH_4_ hydrates which are affected by temperature and pressure [121].

Replacement feasibility is interpreted from kinetic and thermodynamic factors, as well as various forms of CO_2_ [23,102,123,124]. In addition, the difference in the phase equilibrium for methane hydrates and CO_2_ hydrates can decrease the possibility of replacement reactions [121,123]. Figure 5 shows the equilibrium diagram of the CH_4_–CO_2_–H_2_O system [121]. In the diagram, areas A and B lie above the equilibrium curve of H_2_O–hydrate–CO_2_ and below the H_2_O–hydrate–CH_4_ curve. Therefore, theoretically, CH_4_ gas and CO_2_ hydrate can coexist in this area, but it was found that the CO_2_ hydrate is more stable than the CH_4_ hydrate under certain conditions. For example, at 280 K, and a pressure of 2 MPa, CO_2_ can exist as a hydrate, but does not occur in methane. The replacement reactions that occur between CO_2_ replace methane in the clathrate compound allows being conducted at different phase behavior. For the development of hydrate treatment technology, it is necessary to estimate the phase equilibrium data [22,43,46].

Zhou, Long [128] characterized the CH_4_–CO_2_ hydrate using in situ Raman spectroscopy to analyze the dissociation and crystal reformation processes that occur in the replacement of CH_4_–CO_2_ hydrate. The results of the X-ray diffraction analysis showed that the crystal structure of the CH_4_–CO_2_ hydrate mixture was structure 1. This study showed that the hydrate crystal unit collapsed as a single unit, without clear dependence on the gas distribution in the hydrate phase. Moreover, for hydrates containing CH_4_, Raman peaks of CH_4_ and CO_2_ in the hydrate phase showed a transient increase during the hydrate dissociation process, indicating the reformation of the hydrate below the hydrate dissociation surface. According to Ota, Saito [129], there was a replacement of CH_4_–CO_2_ in hydrate CH_4_ with high-pressure CO_2_. These results indicated that the replacement rate was affected by pressure and phase conditions where the driving force was directly influenced by the difference in fugacity of the two guest components, between the fluid and hydrate phases. When CH_4_ hydrate was contacted with CO_2_ under flow conditions, measurements of the hydrate phase indicated the differences in the decomposition rate of cages between medium (M-cage) and small (S-cage) cages in CH_4_ hydrate, with M-cage decomposition is faster than the S-cage. Furthermore, Xu, Cai [22] demonstrated the relationship between total operating pressure and CH_4_ partial pressure in a CH_4_–CO_2_ binary system with a CH_4_–CO_2_ replacement rate and CH_4_ recovery efficiency. On the other hand, the replacement of CH_4_–CO_2_ can occur well when the partial pressure of CH_4_ was lower than the equilibrium pressure for the formation of pure CH_4_ hydrate, and vice-versa for the CO_2_ parameter. In addition, lower partial pressure of CH_4_ leads to a higher replacement rate for a certain pressure condition. It can be concluded that the temperature and pressure conditions constantly change in the replacement process, so these results are important as a reference for the successful CH_4_–CO_2_ replacement. Meanwhile, Ersland, Husebø [130] investigated the replacement process of CH_4_ and CO_2_ by MRI. The process of replacing CH_4_–CO_2_ in the hydrate, without the addition of heat, has potential as a viable strategy for thermodynamically stable long-term CO_2_ sequestration, with the added benefit of associated natural gas production. The MRI proved to provide great information on the spatial distribution of hydrate growth, rate of hydrate formation, and rate of CH_4_–CO_2_ replacement.

For numerical modeling on CO_2_ injected into methane hydrate, Sean, Sato [125] modeled the dissociation process of methane hydrate under-water flow conditions, where the hydrate thermodynamic was stable under pressure and temperature conditions. A relatively low rate of dissociation without the formation of methane bubbles was obtained, where the concentration of methane was dissolved in water and kept below the equilibrium solubility. By combining the experimental results and numerical simulation of flow with the CFD method, the dissociation rate constant was determined based on the ambient flow rate, pressure, and temperature conditions. The results showed that the dissociation process on the surface took place under isothermal conditions. Moreover, Fukumoto, Sean [131] conducted an experiment of CO_2_ hydrate dissociation under-water flow, obtaining hydrates that were thermodynamically stable. In the model, hydrate dissociation was driven by a low CO_2_ mole fraction in water flow. The dissociation rate between the hydrate phase and the aqueous phase was considered as the driving force. As a result, the intrinsic dissociation rate constant of CO_2_ hydrate was established.

Molecular dynamics (MD) simulation is an ideal method to study nucleation at the molecular level since the size of the critical nucleus and formation rate occur on the nanoscale [89,94,106,132,133,134]. According to Bai, Zhang [133], the replacement pathway included the melting of CH_4_ hydrate near the hydrate surface and the subsequent formation of an amorphous CO_2_ hydrate layer. In the dynamic aspect, the replacement process took place near the surface of CH_4_ hydrate relatively easily. However, as the replacement process proceeds, the formation of the amorphous layer of the CO_2_ hydrate provided a significant barrier to the mass transfer of the guest CH_4_ and CO_2_ molecules, which prevented the CH_4_ hydrate from further dissociation and slowed the replacement rate. MD simulations and stabilization energy calculations were carried out to understand the stability of CH_4_ hydrate, CO_2_ hydrate, and CH_4_–CO_2_ hydrate mixture. Hydrate stability decreased with increasing temperature and time [135]. Based on the ratio of stabilization energy of small and large cavities containing CH_4_ and CO_2_, it can be concluded that CO_2_ molecules are suitable for large cavities and vice-versa for CH_4_ molecules [127,136]. In addition, during the growth process, CH_4_ and CO_2_ molecules often replaced each other at certain cage locations adjacent to the hydrate interface [136]. The replacement process occurred during CO_2_ injection into the CH_4_ hydrate reservoir, due to the difference in chemical potential, so the CH_4_ molecule left the hydrate cage, and the empty cage was filled by the CO_2_ molecule [4]. Kossel, Bigalke [137] confirmed that the exchange of guest molecules and gas hydrate dissociations also contributed to the dissociation of CH_4_. Approximately half of the amount of CO_2_ was bound by the exchange of CH_4_ molecules, while the rest was bound by the new CO_2_ hydrates formation.

Other studies [127,133,135,137,138] investigated the dissociation and formation process of methane and carbon dioxide hydrates by MD simulation. Kondori, James [135] evaluated the stability and dissociation of gas hydrate structure 1 for CH_4_–CO_2_ cases using MD simulation, by considering various properties for gas hydrates, such as radial distribution function (RDF), mean square displacement (MSD), lattice parameters, density, potential energy, and molecular diffusion coefficient. Hydrate structures were obtained at different conditions of pressure, temperature, and composition for CH_4_–CO_2_. The result was concluded that the structure with the composition of CH_4_ (25%) + CO_2_ (75%) was stable below 300K at 5MPa. This composition is the best configuration to achieve a stable structure when the carbon dioxide and methane molecules are in large and small cavities, respectively. Furthermore, for the bubble formation and evolution of CH_4_/CO_2_ molecules after dissociation, the size of CH_4_/CO_2_ bubbles is different; however, the shape of both methane and carbon dioxide molecules are almost cylindrical. In addition, Tung, Chen [138] used MD simulations to analyze the replacement that could occur without melting the hydrogen-bonding network of water molecules. Their results suggest that replacement occurs either through a direct exchange of methane and CO_2_ or through the transient co-location of methane and CO_2_ in a single cavity. It was affected by the interfacial distance between the liquid CO_2_ and the solid clathrate hydrate. Based on numerical analysis, it was feasible to replace methane hydrate with CO_2_ in the solid phase without any attempt to alter geological stability.

## 5. Conclusions

Comprehensive research is needed to understand hydrate dissociation mechanisms, hydrate formation, and hydrate recovery conditions in microscale porous media. The aim was to obtain a valid analysis when applying exploration and recovery techniques for gas hydrates, based on the characteristics of the complex physical structures of hydrates in sediment pores, such as multi-phase, multi-component fluid flow, and particle transport. Analyzing dynamic temperatures, concentration distributions, and surface area is effective when evaluating variations in hydrate decompositions, geological characteristics, and flow field limitations of various types. Observations were emphasized on a numerical scale or modeling on a microscale, and compared with experimental studies to obtain precise data. The main points of the development of microscale numerical models are summarized as follows:

1. Microscale devices integrated with modern analytical technologies include Laser Raman spectroscopy (Raman), X-ray diffraction (XRD), X-ray computed tomography (X-CT), scanning electron microscope (SEM), nuclear magnetic resonance (NMR) and high pressure differential scanning calorimetry (DSC).
2. Numerical analysis can be simulated using CFD, Lattice Boltzmann Method (LBM), and molecular dynamics (MD). MD simulations consist of the radial distribution function (RDF), mean square displacement (MSD), thermal expansion coefficient, diffusion coefficient, capacity of heat, and thermal conductivity for various gas hydrate structures under particular thermodynamic conditions.
3. Simultaneous analysis of hydrate dynamic numerical models is performed for temperature, concentration, porosity, saturation rate, and permeability under different hydrate formation coatings (sand coating, bridging, or spacing).
4. The gas hydrate exploitation process not only focuses on the formation, dissociation, and recovery processes, but also considers the blockage and leakage of hydrates.
5. Dynamic analysis of impurities, such as salinity and mud, could be added to bulk fluid; it can specifically correspond to the following:
  - Analysis of the effective surface area of the gas hydrate in the formation attached to the sandstone in different forms.
  - Analysis of the influence of factors related to the pore size distribution of mineral particles and hydrates on permeability changes in porous media.
  - Considering the formation characteristics of different hydrates, particle coating, and pore-filling; the relationship between relative permeability and hydrate saturation is proposed.
  - In the process of hydrate conversion, gas hydrate is transformed into water phase hydrate. The hydration saturation increases with the increase of relative porosity, relative pore size, and relative particle size. The hydrate will reduce the porosity due to the pore space occupied by the hydrate. Then, the permeability decreases with the increase of the hydrate phase in porous sediments.
  - In the pore space with capillary pores, micromorphic hydrates occur spontaneously, and the permeability decreases approximately linearly with the increase of hydrate saturation during the nucleation process of the crystal grain surface; the permeability is often higher than predicted by previous analysis model values.
  - In the transition zone simulation, the downward trend of permeability is converted from the particle coverage model to the pore filling model, and the tortuosity and surface area are analyzed to understand the mechanism of permeability reduction.
  - Mud erosion is a result of the mud surface with tremendous shear stress; a moderately wide space appears in the pores of the sand particles, which slows down the water flow to less than the critical value.

## Data Availability

The data used to support the findings of this study are included within the article.
